# Ribosome Protection Proteins—“New” Players in the Global Arms Race with Antibiotic-Resistant Pathogens

**DOI:** 10.3390/ijms22105356

**Published:** 2021-05-19

**Authors:** Rya Ero, Xin-Fu Yan, Yong-Gui Gao

**Affiliations:** 1 Department of Molecular Biology, Institute of Molecular and Cell Biology, University of Tartu, 51010 Tartu, Estonia; 2 School of Biological Sciences, Nanyang Technological University, Singapore 637551, Singapore; xfyan@ntu.edu.sg; 3 NTU Institute of Structural Biology, Nanyang Technological University, Singapore 639798, Singapore

**Keywords:** antibiotic resistance, ribosome protection, ABC-F proteins, novel antibiotics

## Abstract

Bacteria have evolved an array of mechanisms enabling them to resist the inhibitory effect of antibiotics, a significant proportion of which target the ribosome. Indeed, resistance mechanisms have been identified for nearly every antibiotic that is currently used in clinical practice. With the ever-increasing list of multi-drug-resistant pathogens and very few novel antibiotics in the pharmaceutical pipeline, treatable infections are likely to become life-threatening once again. Most of the prevalent resistance mechanisms are well understood and their clinical significance is recognized. In contrast, ribosome protection protein-mediated resistance has flown under the radar for a long time and has been considered a minor factor in the clinical setting. Not until the recent discovery of the ATP-binding cassette family F protein-mediated resistance in an extensive list of human pathogens has the significance of ribosome protection proteins been truly appreciated. Understanding the underlying resistance mechanism has the potential to guide the development of novel therapeutic approaches to evade or overcome the resistance. In this review, we discuss the latest developments regarding ribosome protection proteins focusing on the current antimicrobial arsenal and pharmaceutical pipeline as well as potential implications for the future of fighting bacterial infections in the time of “superbugs.”

## 1. Ribosome-Targeting Antibiotics and Resistance in Clinical Practice

Ribosome is one of the most conserved and sophisticated macromolecular machines that carries out the essential process of protein synthesis in cells. All ribosomes consist of two subunits, each assembled from one or more ribosomal RNA (rRNA) molecules as well as numerous ribosomal proteins (r-proteins). The decoding center (DC) of the smaller subunit (30S in bacteria) is responsible for decoding the genetic information delivered by messenger RNA (mRNA). However, it is the rRNA of the larger subunit (50S in bacteria) that carries the main catalytic function of the ribosome—the formation of the peptide bond between the incoming amino acid attached to the transfer RNA (tRNA) in the aminoacyl- (A) site and the nascent polypeptide attached to the tRNA in the peptidyl- (P) site. In principle, the ribosome functions as an entropy trap by precisely positioning the tRNA substrates for peptide bond formation [1]. The region of the large subunit (made up of nucleosides from 23S rRNA domain V in prokaryotes) involved in the latter is called the peptidyl transferase center (PTC). Adjacent to the PTC is the opening of the tunnel through which the nascent peptide exits the ribosome.

Given the fundamental nature of protein synthesis (a.k.a. translation), it is not surprising that the ribosome is one of the main targets for chemical agents produced by certain microorganisms to give them an edge over other microorganisms in nature [2,3,4]. Furthermore, the features characteristic of bacterial translation apparatus have been historically exploited for the selection of natural antimicrobials and the development of synthetic drugs to combat bacterial infections in human and veterinary medicine as well as to benefit agriculture and food production. The discovery of antibiotics (AB) has been regarded as one of the most significant achievements in modern medicine, saving countless lives and enabling important medical procedures, including surgery and chemotherapy [5].

Unfortunately, almost as quickly as AB are adopted in clinical practice, they are in danger of becoming antiquated due to pathogens acquiring distinctive antibiotic resistance (ARE) mechanisms that have evolved in bacteria to protect themselves from “chemical weaponry” produced by themselves or rival microorganisms [2]. ARE genes can easily spread between organisms in the presence of sufficient pressure such as over- and misuse of AB in human and veterinary medicine as well as farming and food production. For instance, the AB-producing soil bacteria *actinomycetes* are suspected to be the origin of ARE in many other bacterial species and evidence for the exchange of ARE determinants between soil bacteria and clinical pathogens has been reported [3,6]. Therefore, it is not surprising that while there are currently numerous classes of chemically diverse AB in clinical practice that interfere with protein synthesis by binding to the ribosome (Table 1), there is an imminent threat of majority, if not all of them, being rendered obsolete due to the emergence and spread of ARE among human pathogens. Indeed, ARE mechanisms have been identified for nearly every AB currently in use in clinical practice, including virtually every ribosome-targeting AB. Table 1 summarizes the major classes of bacterial translation inhibitors termed critically important antimicrobials for human medicine (considering existing and potential ARE) by the World Health Organization (WHO) as of 2018 [7]. The WHO classification is based on AB being the sole, or one of the limited available therapies, to treat serious infections caused by pathogens that may acquire ARE genes from non-human sources in order to emphasize the importance of their appropriate use.

Macrolides and ketolides are a class of ribosome-targeting drugs that bind to the 50S nascent peptide exit tunnel (NPET) adjacent to PTC and cause ribosome stalling when specific amino acid motifs are encountered at PTC and nascent chain progression is hindered [8,9]. Thus, macrolides and ketolides should be considered context-specific (depending on the nature of the nascent chain and the structure of the drug) modulators of protein synthesis. Macrolides and ketolides (foremost azithromycin, erythromycin, and telithromycin) are classified as the highest-priority clinically important microbials [7] (Table 1). Azithromycin (first or second choice treatment for chlamydia, cholera, gonorrhea, and bacterial diarrhea/dysentery) and clarithromycin (first or second choice treatment for severe pneumonia and pharyngitis) are included in the “watch group” in the WHO’s 2019 Model List of Essential Medicines naming the safest and most effective medicines needed in the healthcare system [10]. “Watch group” includes AB with a relatively high risk for the selection of ARE, whose usage should therefore be monitored and restricted to essential first or second choice empiric treatment options for a limited number of specific infections. Macrolides are one of the few available therapies for serious *Campylobacter* infections (particularly in children) and limited therapy for multi-drug-resistant (MDR) *Salmonella* and *Shigella* infections [7]. The emergence of ARE to macrolides has led to the development of telithromycin, which is the first clinically prescribed ketolide against macrolide-resistant strains but is rarely used because of a restricted label and liver toxicity warnings.

WHO’s high-priority critically important microbials [7] include aminoglycosides (e.g., amikacin and gentamicin), oxazolidinones (linezolid), and the tuberactinomycin capreomycin (Table 1). Aminoglycosides bind to the 30S DC region and inhibit the translocation step of elongation as well as increase the error rate [2]. Amikacin (second choice for sepsis in neonates and children) and gentamicin (first or second choice for severe pneumonia, sepsis in neonates and children, gonorrhea, and surgical prophylaxis) are listed as the WHO “access group” AB and have activity against a wide range of commonly encountered susceptible pathogens, show lower ARE potential, and are therefore recommended as essential first or second choice empiric treatment options that should be widely available, affordable, and quality assured [7]. However, aminoglycoside clinical usage has several limitations. All aminoglycosides can cause irreversible vestibular and auditory toxicity and may affect renal function [11]. Neomycin and kanamycin are limited to topical use in small amounts due to toxicity. Aminoglycosides often require intravenous administration (not well absorbed orally) and are infrequently used alone but rather used in combination with other classes of AB in order to address ARE. Aminoglycosides are the sole or a limited therapy as part of the treatment of *enterococcal* (ARE to aminoglycosides not uncommon) infections, MDR tuberculosis, and MDR *Enterobacteriaceae*. Plazomicin (approved for medical use in the United States in 2018 and sold under the brand name Zemdri), used to treat complicated urinary tract infections, is classified by the WHO as “reserve group” AB that should be reserved for the treatment of confirmed or suspected infections due to MDR pathogens and treated as “last resort” when no alternatives are available [10]. In addition to plazomicin, the “reserve group” includes the oxazolidinone linezolid whose application should also be restricted to highly specific patients and settings, when all alternatives have failed or are not suitable. In general, the “reserve group” AB usage should be monitored and reported to preserve their effectiveness in avoiding ARE emergence and spread. Further highlighting the urgency of ARE is the fact that there is a high absolute number of people affected by diseases for which either macrolides or linezolid is the sole or one of the few therapies available. Unlike macrolides that bind to NPET, oxazolidinones bind to 50S PTC A-site but also interfere with protein synthesis in a context-dependent manner, leading to unproductive binding–dissociation cycles of incoming aminoacyl-tRNAs [8,12,13]. Linezolid (the first approved oxazolidinone) has been in clinical use since 2000 and is a limited therapy for infections due to MDR *Enterococcus* and methicillin-resistant *Staphylococcus aureus* (MRSA). MRSA is a major cause of morbidity and mortality worldwide, often requiring long and costly hospital stays, and is therefore considered a serious threat by the Centers for Disease Control and Prevention (CDC). The last class of AB under the WHO’s high-priority critically important antimicrobials is ribosome subunit interface binding tuberactinomycin, which inhibits translocation and whose representative capreomycin is used in combination with other AB solely for the treatment of drug-resistant tuberculosis and other mycobacterial infections [7] (Table 1).

Moving on, phenicols (chloramphenicol and thiamphenicol), lincosamides (clindamycin and lincomycin), steroid antimicrobials (fusidic acid), streptogramins (quinupristin and dalfopristin), and tetracyclines (doxycycline) are classified as highly important antimicrobials by the WHO [7]. Like oxazolidinones, phenicols bind to PTC A-site and interfere with aminoacyl-tRNA positioning in a context-dependent manner influenced by the nature of the amino acid-forming peptide bonds in the PTC [8,12]. Therefore, phenicols cannot be considered universal inhibitors of protein synthesis, but rather modulators. Chloramphenicol is the WHO “access group” AB and one of the limited therapies for acute bacterial meningitis, typhoid, and non-typhoid fever, and respiratory infections [7,10]. Lincosamides bind to PTC A-site as well and interfere with aminoacyl-tRNA accommodation [14,15]. Clindamycin is the WHO “access group” AB used for the treatment of several bacterial infections, including strep throat, pneumonia, middle ear infections, and endocarditis. It can also be used to treat some cases of MRSA, but the WHO notes the risks of ARE [7,10]. Streptogramins include two structurally and functionally distinct subclasses: group A (S_A_) bind to PTC overlapping A- and P-sites, thereby inhibiting peptide bond formation; and group B (S_B_) bind to NPET, thereby hampering the egress of the nascent chain [9]. S_A_ and S_B_ act synergistically, with S_A_ binding promoting the binding of S_B_. Streptogramins have been used as livestock feed additives for decades but were not approved by the Food and Drug Administration (FDA) until the introduction of quinupristin (S_B_)-dalfopristin (S_A_) (Synercid) in 1999. The clinical use of this combination therapy is limited by its intravenous administration as well as narrow spectrum of activity and is therefore reserved for hospitalized patients with MDR skin infections or with bacteremia caused by vancomycin-resistant *Enterococcus faecium*. Synercid is active against MRSA but ARE may result from transmission from non-human sources [7]. Fusidic acid (FA) inhibits protein synthesis by binding to elongation factor G (EF-G) on the ribosome and preventing the disassembly of the post-translocation complex; the resultant steric occlusion of the A-site by EF-G blocks the delivery of incoming aminoacyl-tRNA [16,17]. FA is the sole or limited therapy for MRSA infections; unfortunately, ARE among clinical isolates of *S. aureus* has increased dramatically in recent years [18]. Tetracyclines target the 30S DC A-site and inhibit the delivery of aminoacylated tRNA in the A-site. The WHO “access group” doxycycline is used to treat pneumonia, Lyme disease, cholera, typhus, and syphilis, among other infections, and is a limited therapy for infections due to *Brucella*, *Chlamydia*, and *Rickettsia* [7]. Finally, pleuromutilins are considered by the WHO as important antimicrobials [7] (Table 1). Pleuromutilins interact with 50S PTC A- and P-site, hindering proper positioning of tRNAs and leading to the inhibition of protein synthesis, especially at initiation codons [19]. Pleuromutilins are highly potent drugs against MDR Gram-positive and some Gram-negative bacteria [20] used in veterinary medicine and since 2007 as topical treatment in humans (retapamulin). The potential for ARE development in the clinic is predicted to be slow as confirmed by extremely low ARE rates to this class in animal infections despite the use of pleuromutilins in veterinary medicine for over 30 years [20].

The ribosome binding mode and translation inhibition mechanism of the ribosome-targeting AB classes, as well as the various ARE mechanisms (see Table 1) adopted by bacteria to overcome them, have been covered in great detail in many excellent reviews [2,3,4,18,21,22]. In short, despite the large size of the ribosome, relatively few functionally important regions (foremost PTC/NPET and DC) are targeted by the current arsenal of clinically relevant AB, which results in significant overlap between many of the binding sites. PTC-targeting AB binding sites overlap with the A-site tRNA (e.g., phenicols, lincosamides, and oxazolidinones) or span both A- and P-sites (pleuromutilins and S_A_). The binding sites of the macrolides and S_B_ classes are located adjacent to the PTC within the NPET through which the growing polypeptide chain transverses during translation. Most macrolide and S_B_ members do not inhibit peptide bond formation per se but rather prevent elongation of most nascent chains, which leads to peptidyl-tRNA drop-off and abortion of translation, resulting in imbalance in protein production. As mentioned, ARE mechanisms have been identified for virtually every ribosome-targeting AB (Table 1). Most of the prevalent ARE mechanisms are relatively well understood and their clinical significance has been recognized. These include the mutation or modification of the target sites in the ribosome to reduce or abolish AB binding. Alternatively, AB themselves can be degraded, modified, or pumped out of the cell by dedicated enzymes, thereby lowering the intracellular concentration to non-toxic levels [3,21,22]. In this review, we focus on the less well known but by no means less significant ARE mechanism mediated by ribosome protection proteins. This ARE mechanism has only recently stepped into the limelight with respect to a detailed understanding of the underlying mechanism, prevalence in human pathogens, as well as potential impact on diverse classes of AB used for combating common microbial infections in the era of MDR “superbugs”.

## 2. Ribosome Protection Proteins

Ribosome protection constitutes a ribosome-interacting factor-assisted target protection mechanism [18]. Unlike target alteration (AB binding site mutation or modification, e.g., ribosome methylation by specialized methyltransferases), target protection does not involve a permanent change to the target. In case of a target as conserved and functionally fine-tuned as the ribosome, permanent changes that alter the target in order to reduce or abolish AB binding are often accompanied by a fitness cost due to reduced functionality of the highly conserved centers of the ribosome [23]. Hence, there tends to be a trade-off between optimal fitness and ARE. For example, to overcome the fitness cost resulting from the methylation of 23S rRNA residue A2058 that confers resistance to macrolides, ketolides, lincosamides, and S_B_, the corresponding Erm methyltransferases are not expressed constitutively but are induced only in the presence of corresponding AB [24]. This inducible system allows *S. aureus* to survive in the presence of an AB yet still maintain optimal growth when conditions are favorable [23]. In contrast, ribosome protection results from persistent or repeated direct physical interaction between ribosome and specialized ribosome protection proteins (RPP) that does not introduce a permanent change to the ribosome in order to rescue the translation apparatus from AB inhibition. Target protection had previously not been considered a leading cause of ARE in the clinical setting; however, it has recently become evident that it can cause ARE to a vast majority of the clinically relevant translation inhibitors (Table 1). Currently, three classes of RPP have been identified: Tet-type proteins, which mediate ARE to tetracycline; Fus-type proteins, which mediate ARE to FA; and the most recent class to emerge—ABC-F proteins—which mediate resistance to diverse AB, including macrolides, oxazolidinones, phenicols, lincosamides, streptogramins, and pleuromutilins.

### 2.1. Tet-Type RPP

Due to the relative lack of major side effects and cheap cost, tetracyclines (TET) have been used extensively in the treatment of various infections in humans as well as growth promotors in agriculture, resulting in widespread ARE among clinically relevant pathogens [25]. Members of the TET class AB bind in a position overlapping with the A-site of DC in 30S and inhibit translation by interfering with the delivery of the incoming amino-acyl-tRNA by elongation factor Tu (EF-Tu) during translation elongation [26,27]. This was the first class of AB for which RPP were identified in the early 1990s [28] but the Tet-type RPP have only recently come to public attention as a significant contributor of ARE in human pathogens [18,25]. TetM and TetO are the most well known of the 15 classes of Tet-type RPP currently listed in the Comprehensive Antibiotic Resistance Database [29,30]. TetM is the most prevalent TET ARE determinant in clinical isolates of *streptococci*, *staphylococci*, as well as *enterococci* [18]. The TetO gene has been described in *Campylobacter*, *Streptococcus*, and *Enterococcus* species. Collectively, Tet-type RPP are found in a diverse range of Gram-negative and -positive pathogens, representing the major cause of ARE in the latter. Phylogenetic studies have revealed one distinct branch of TET RPP suggesting a single ancient point of origin from duplication of an elongation factor G-like gene [18]. Horizontal transmission is the main way to spread RPP-mediated ARE among bacteria (e.g., the *tetM* gene is found in various transposons) [25,30].

**Figure 1:**
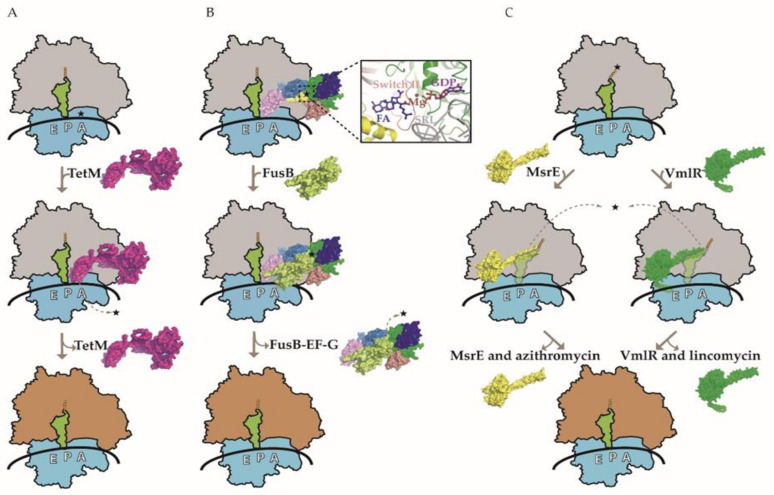
Three models of ribosome protection protein-mediated antibiotic resistance. (**A**) A model for ribosome protection against tetracycline (TET) mediated by the TetM protein. Drug-stalled ribosome with tRNA in the P-site (green) is rescued by TetM (pink), which competes with TET (shown with star) in the A-site, thereby purging it from the ribosome. The subsequent GTP hydrolysis-dependent release of TetM from the ribosome enables protein synthesis to resume. (**B**) A model for ribosome protection against fusidic acid (FA) mediated by the FusB protein. FA interaction with elongation factor G (EF-G) prevents its dissociation from the ribosome. FusB (lime green) interacts with the ribosome-bound EF-G, leading to its release and allowing translation to proceed in the presence of FA. The domains G, G’, II, III, IV, and V of EF-G are colored green, blue, deep salmon, yellow, and sky blue, respectively. An enlarged view of the FA binding pocket is shown, involving EF-G domains G, II, and III. EF-G switch II (residues 82–102) is colored red and the 23S ribosomal RNA sarcin-ricin loop (SRL) is colored gray. In addition, GDP and Mg^2+^ in the vicinity of FA are also shown. Notably, FusB does not interact with the same region of EF-G as FA and there is no evidence for direct physical displacement of the drug. (**C**) A model for ribosomes protection against various classes of PTC/NPET-targeting AB mediated by the ARE ABC-F proteins. Two representatives of ARE ABC-F proteins, MsrE (yellow) and VmlR (dark green), are shown to bind to the E-site of the drug-stalled ribosome. Their antibiotic resistance domain (ARD) distorts the tRNA in the P-site (green) in order to access the drug-binding site. Allosteric and/or steric interactions in PTC promote the dissociation of drugs. ATP binding may promote RPP–ribosome interaction, while ATP hydrolysis leads to the dissociation of RPP from the ribosome. Drugs corresponding to MsrE and VmlR are azithromycin and lincomycin, respectively (also shown with stars). Ribosomes with gray and orange large subunits represent translationally inactive and active complexes, respectively.

TetM and TetO catalyze the GTP-dependent release of TET from the ribosome (Figure 1A) and share a significant sequence and structural similarity with elongation factors G (EF-G) and Tu (EF-Tu) [31,32]. Cryo-electron microscopic (cryo-EM) studies indicate that Tet-type RPP have overlapping binding sites with EF-G as well as TET in the ribosome A-site [33,34], implying that ARE is mediated through direct physical displacement of the AB. Indeed, conserved proline (Pro-509) of TetM is located directly within the TET-binding site, where it interacts with the 30S 16S rRNA nucleotide C1054 [35]. As RPP becomes trapped on the ribosome in the presence of the non-hydrolyzable GTP analog, it appears that GTP hydrolysis is required for RPP dissociation rather than AB release. Conformational changes within RPP that are associated with GTP hydrolysis may not only facilitate its dissociation from the ribosome but also induce a persistent conformational change within the AB binding site. These conformational changes can likely hinder the immediate rebinding of the AB as well as promote the subsequent delivery of the aminoacyl-tRNA by EF-Tu and enable the translation to continue in the presence of TET [26,33]. Notably, RPP confer ARE to some (tetracycline, minocycline, and doxycycline) but not all TET classes of AB. For instance, tigecycline, eravacycline, and omadacycline retain translational inhibition in the presence of RPP [25]. This can be potentially attributed to the bulky side chains at the C9 position of the D-ring in these TET derivatives that mediate the AB ribosome binding mode and affinity, thereby likely rendering RPP incapable of dislodging the drugs [27,36,37]. However, the exact mechanism of RPP evasion is not fully understood and *E. faecalis* resistant to tigecycline due to transposon-encoded constitutively expressed TetM has been reported [38].

### 2.2. Fus-Type RPP

Fusidic acid (FA) is widely used as a topical treatment of staphylococcal skin infections and is one of the few remaining AB that can be used to treat MRSA orally. Unfortunately, ARE to FA among clinical isolates of MRSA and other *staphylococci* has increased dramatically over the last few decades [39]. Clinical ARE predominantly results from horizontal acquisition of Fus-type RPP genes that seem to originate from the duplication of accessory translation factors, which have obtained ARE activity during evolution [40,41,42].

FA acts by interfering with EF-G functioning during translation. Once translocation has occurred, EF-G dissociates from the ribosome vacating the A-site for the incoming aminoacyl-tRNA. In the presence of FA, the drug binds to EF-G on the ribosome and inhibits its release, thereby preventing disassembly of the post-translocation complex and blocking the delivery of incoming aminoacyl-tRNA [16,43]. The small two-domain metalloprotein FusB is the most studied FA RPP. FusB binds to and promotes the dissociation of FA-trapped EF-G from the ribosome, allowing translation and/or ribosome recycling to resume in the presence of the drug [42,44,45] (Figure 1B). In contrast to other RPP, Fus proteins do not interact with its target in the proximity of the bound AB. More specifically, Fus protein makes contacts with domain IV and V of ribosome-bound EF-G, whereas FA binds to a pocket between domains G and III [16,40]. There is no evidence for direct physical displacement of AB by FusB. Instead, Fus-type RPP negate the EF-G ribosome tethering effect of FA by inducing conformational changes in EF-G domains IV and V as well as the dynamics of domain III, enabling EF-G dissociation from the ribosome [40]. In other words, Fus-type protein-mediated modulation of EF-G function can overcome FA inhibition, resulting in ARE. FA is likely to dissociate from free EF-G due to low affinity.

### 2.3. ABC-F Subfamily RPP

ATP-binding cassette subfamily F (ABC-F) proteins first gained attention as ARE-mediating RPP about 5 years ago when Sharkey et al. showed that purified ABC-F proteins (*S. aureus* VgaA and *E. faecalis* LsaA) are capable of displacing AB (S_A_ virginiamycin M and lincomycin, respectively) from the ribosome and rescuing translation in vitro [46]. These experiments provided the first direct evidence to the hypothesis that ABC-F proteins mediate ARE through target protection [47]. Since then, it has become apparent that ABC-F represents a widespread family of proteins that collectively provide ARE to a broader range of clinically important AB classes than any other group of ARE proteins. Indeed, ABC-F members constitute a major source of clinically significant ARE to almost all classes of PTC/NPET-targeting AB (including macrolides, oxazolidinones, phenicols, lincosamides, streptogramins A and B, as well as pleuromutilins) [48,49,50,51] (Table 1).

In-depth study of ABC-F subfamily members across all species with sequenced genomes revealed that, unlike Tet- and Fus-type RPP, known ARE ABC-F proteins are not confined to a distinct phylogenetic lineage [18,51]. Instead, numerous phylogenetic lineages (ARE 1-8 in Table 2) exist suggesting that ARE has arisen on several occasions among ABC-F of unknown function (e.g., potential translation factors). ARE emergence notably benefits the cell and is likely to be retained during evolution, especially if it comes without the loss of cellular fitness. As yet uncharacterized bacterial ABC-F subfamily members cluster with known groups of ARE ABC-F proteins, it seems likely that additional members of the ABC-F mediating clinically relevant ARE remain to be discovered [51]. Furthermore, non-ARE ABC-F protein can readily evolve to gain ARE function given sufficient pressure from mis- and overuse of AB [52].

Extensive phylogenetic studies have determined that ARE ABC-F members are widespread in the chromosomes of Gram-positive and, to a lesser extent, Gram-negative bacteria, as well as in mobile genetic elements of many clinically isolated pathogens [48,51,52,53,54,55]. These include the ESKAPE species (*Enterococcus faecium*, *Staphylococcus aureus*, *Klebsiella pneumoniae*, *Acinetobacter baumannii*, *Pseudomonas aeruginosa*, and *Enterobacter* species) that contribute to a substantial portion of hospital-acquired MDR infections. Notably, ABC-F (e.g., the *optrA* and *poxtA* genes) mediate ARE against the clinically crucial AB linezolid (Table 1), which is used in the treatment of MRSA nosocomial strains and vancomycin-resistant *Enterococcus faecium* [56]. Moreover, the *optrA* gene, whose presence has also been reported in other *staphylococci*, is to date the only known horizontally transmissible determinant capable of conferring ARE to tedizolid, a second-generation oxazolidinone approved by the FDA in 2014 [57]. *Pseudomonas aeruginosa* (*P. aeruginosa*) is a common Gram-negative pathogen whose infections have become increasingly severe due to the spread of MDR to various AB, especially β-lactams, aminoglycosides, quinolones, and sulfonamides, resulting in very limited treatment options [58]. Indeed, carbapenem-resistant *P. aureus* ranks 2nd (following carbapenem-resistant *A. baumannii*; the infamous MRSA ranks 12th) in the final ranking of the WHO’s 2017 “Prioritization of Pathogens to Guide Discovery, Research and Development of New Antibiotics for Drug-Resistant Bacterial Infections, Including Tuberculosis” [59]. Macrolides have been used to treat MDR *P. aeruginosa* infections; however, due to increasing applications in clinical practice, MsrE-mediated ARE to macrolides has started to spread worldwide [60,61]. MsrD protein plays a predominant role in conferring macrolide ARE to *Streptococcus pneumoniae* and *Streptococcus pyogenes* isolates in various parts of the world, including the US and the UK [62]. In *staphylococci*, msr-type RPP (particularly MsrA) are responsible for ARE in up to 30% of the strains exhibiting the MKS_B_ (ARE to macrolides, ketolides, and S_B_) phenotype [48]. ABC-F is also a major contributor to pleuromutilin ARE in *staphylococci* as the *vga* genes account for all instances of ARE to retapamulin in the nearly 6000 *S. aureus* isolates tested [63]. While altogether the incidence of Vga-mediated pleuromutilin ARE in human *S. aureus* isolates is considerably lower than in isolates from animals (where it has spread due to extensive use of pleuromutilins in food production and agriculture), this is very likely to change in the future in response to the recent (2019 in the US and 2020 in Europe) approval of the first systemic pleuromutilin lefamulin (sold under the brand name Xenleta) in human medicine [64,65]. ABC-F genes can disseminate easily from strain to strain via MDR conferring plasmids, and many examples of horizontal gene transfer have been reported [66]. Consequently, ABC-F family proteins can be an important source of ARE in “superbugs.”

No individual ABC-F protein confers ARE to all of the PTC/NPET-targeting AB. Three distinct profiles can be distinguished in clinical isolates: the MKS_B_ phenotype conferring ARE to macrolides, ketolides, and S_B_ (e.g., Msr proteins); the PLS_A_ phenotype conferring ARE to pleuromutilins, lincosamides, and S_A_ (multiple variant proteins arising from distinct bacterial lineages, e.g., Vga and Lsa proteins); and the Pho phenotype conferring ARE to phenicols and oxazolidinones (e.g., OptrA and PoxtA) [18,51] (Table 2). ARE 4 and ARE 5 phylogenetic lineage members provide self-protection against various classes of drugs in AB-producing bacteria such as *Streptomyces*. Notably, cross-resistance mediated by individual ARE ABC-F proteins to different AB correlates with spatial overlap of AB binding sites (Table 2). This phenomenon has become better understood in recent years following the structural and functional characterization of the ribosome protection mechanism of several members of the ARE ABC-F proteins.

ABC-F proteins consist of two tandem nucleotide binding domains (NBD) in a single polypeptide chain connected by a 60–100-residue linker (known as the ARD for antibiotic resistance domain or the PtIM for P-site tRNA interaction motif) that is the defining feature of the ARE ABC-F family [46,48,51]. The ARD forms an α-helical hairpin containing an inter-helical loop of variable length [49,67,68]. The ARD inter-helical loop differs considerably in length and sequence among the ARE ABC-F proteins, and alterations within this sequence can alter the AB specificity [18,46,49]. ARE ABC-F proteins may include an “arm” subdomain within the NBD1 as well as an additional C-terminal extension (CTE). The first structural insight into how ABC-F proteins confer ARE came from the cryo-EM structure of *P. aeruginosa* macrolide resistance protein MsrE bound to the *T. thermophilus* ribosome with a cognate deacylated tRNA in the P-site [67]. MsrE protein was trapped on the ribosome by using a non-hydrolysable ATP homolog. Shortly thereafter, the cryo-EM structure of *Bacillus subtilis* (*B. subtilis*) lincomycin and S_A_ resistance-associated ATPase-deficient VmlR protein in complex with the ErmDL-stalled *B. subtilis* ribosome was reported [68]. Several recent reviews have compared the two structures in detail [49,50,52] and a common theme of the RPP mechanism is emerging (Figure 1C). Namely, the substrate is the AB-stalled ribosome and the RPP bind in the vacant exit (E) site in the ATP form adopting the closed conformation with the ARD reaching deeper into the ribosome towards the PTC/NPET region. In both the MsrE and VmlR structures, the P-tRNA interacts with the RPP, resulting in its notable shift toward the E-site, while the acceptor stem is shifted away from the PTC toward a site usually occupied by the acceptor stem of the fully accommodated A-tRNA [67,68]. RPP likely stabilizes the P-tRNA to prevent its drop-off. These conformational changes of P-tRNA allow the ARD to reach PTC in the VmlR structure as well as the adjacent NPET region in the MsrE structure. It should be noted that there is a correlation between the length of the inter-helical loop, its positioning in the ribosome, and the corresponding AB binding site. VmlR has a shorter loop and confers ARE to lincosamides, S_A_, and pleuromutilins, which target PTC A-site overlapping with P-site and NPET, while MsrE has a notably longer loop that projects deeper into NPET where the macrolides bind. MsrE ARD loop leucine residue (Leu-242) comes within 1.8 Å and could clash with the ribosome-bound macrolides and S_B_ [67]. Substituting this leucine with alanine leads to a near-complete loss of MsrE’s ability to mediate azithromycin (AZM) ARE, which also suggests a steric component to drug release [67]. In addition, conformational changes in the PTC region and a slight widening of the NPET around the macrolide binding site are observed in the MsrE-ribosome structure. Similar conformational changes in the PTC region are observed in the VmlR-ribosome structure; however, direct steric interference between the RPP and AB seems not critical based on the cryo-EM structure as well as mutagenesis studies of the VmlR phenylalanine (Phe-237) residue that comes closest to the bound AB [68]. Curiously, neither MsrE nor VmlR confer resistance to oxazolidinones and phenicols, even though their binding sites on the ribosome overlap. Furthermore, it is not clear how OptrA and PoxtA manage to dislodge oxazolidinones and phenicols from the ribosome as these proteins have a relatively short ARD that is not expected to reach into the PTC where the corresponding AB bind [18].

The ATPase activity of ABC-F is critical for ARE [49,67,69]. ATP hydrolysis does not seem to be required for AB release per se; instead it likely drives the two NBD domains apart triggering the release of RPP from the ribosome so that translation can resume. Murina et al. have shown that VgaA can hydrolyze other NTPs as well and operates as a molecular machine requiring NTP hydrolysis (not just NTP binding) for ARE [69]. Persisting allosteric changes in the ribosome (as is the case following Tet-mediated TET release discussed previously), tRNA (re)-accommodation into PTC A- and P-sites or nascent chain settlement into NPET (i.e., following macrolide release) likely block AB rebinding. Furthermore, AB displacement activity could be coupled to functional partners that degrade or pump AB out of the cell. Although continual dynamic displacement of AB driven by RPP ATPase activity might be required to “plow through” the stalling-prone sequences of certain nascent chains in the presence of the context-dependent translation inhibitors (macrolide, oxazolidinones, and phenicols), RPP rebinding is likely hindered by deacylated tRNA progression into the E-site when AB no longer poses an issue.

Taken together, comparison of the RPP from different ABC-F phylogenetic lineages and with different ARE profiles suggests a common yet adaptable mechanism of ARE to translation elongation inhibitors, which trap the ribosome with the tRNA in the P-site, resulting in slow or stalled translation. AB displacement is achieved by a combination of direct interactions of RPP with the drug as well as by ARD loop-mediated allosteric relay of changes to the PTC and/or NPET in the vicinity of the AB binding site by altering the orientation of rRNA residues involved in AB binding.

## 3. Development of Novel Antibiotics

### 3.1. Pharmaceutical Pipeline and Implications for Future

Since 2017, only three new AB targeting the translation apparatus (the aminoglycoside plazomicin, the tetracycline omadacycline, and the pleuromutilin lefamulin) have been approved by the FDA in the US (Table 3). These AB improve the treatment options for infections caused by the WHO’s highlighted MDR pathogens: carbapenem-resistant *A. baumannii* (CRAB), carbapenem-resistant *Enterococcus species* (CRE), and methicillin-resistant *S. aureus* (MRSA) [59]. However, only lefamulin can be considered a novel (new chemical class with a novel mode of action) AB in human medicine. While pleuromutilins have been listed by the WHO as important antimicrobials [7] (Table 1) with highly potent activity against MDR Gram-positive and some Gram-negative bacteria, until now they have been used mostly in veterinary medicine and only as a topical treatment of skin infections in humans (retapalulin). Furthermore, as mentioned previously, the development of ARE against pleuromutilins in the clinic is predicted to be slow based on very low ARE rates in animals despite the extensive use of the pleuromutilins tiamulin and valnemulin in veterinary medicine for decades [17]. However, the spread of ARE ABC-F genes (e.g., *vgaA*) in pathogens such as MRSA is likely to increase, thereby threatening the utility of pleuromutilins in the long run. While pleuromutilins require prudent use and monitoring in human and veterinary medicine, this class presents a good candidate for future AB development. Yet there does not appear to be any pleuromutilins in the pharmaceutical pipeline according to the WHO and Pew Charitable Trust as of 31 December 2020 (Table 3) [70,71].

The omadacycline (recently approved for clinical use) and eravacycline (currently in phase 3 clinical trials) derivatives of tetracycline are not subjected to Tet-type RPP-mediated ARE. Though the exact reason for this is unknown, it is likely attributed to the novel moieties at the C9 position of these drugs compared to the parent tetracycline [59,72,73]. The observation that there are several other tetracycline derivatives in phase 1 of the current pharmaceutical pipeline (Table 3) suggests an increased interest in developing this class of AB to target the clinically relevant MDR pathogens. Linezolid was the first oxazolidinone class of AB approved for clinical use in humans in 2000, followed by the second-generation oxazolidinone tedizolid in 2014. To a certain degree, tedizolid helped to overcome the ARE that appeared (e.g., in vancomycin-resistant *enterococci*) and started to spread shortly after the broad use of linezolid. However, while tedizolid activity is not affected by ribosome methylation (e.g., presence of the plasmid-encoded *cfr* gene)-mediated ARE, it is targeted by ribosome protection via plasmid-encoded OptrA, albeit to a lesser extent than linezolid [57]. The lesser effect of OptrA is likely due to the higher ribosome affinity of tedizolid (compared with linezolid) and implies that the ribosome protection mechanism could potentially be negated with sufficiently potent oxazolidinones [57]. There are currently two oxazolidinones in the pharmaceutical pipeline (contezolid and delpazolid in phase 3 and phase 2 clinical trials, respectively); however, they are not expected to have activity against the CDC urgent or WHO critical threat pathogen or MRSA, and their resistance/susceptibility to RPP has not been reported (Table 3). The latter also applies to the phase 2 oxazolidinone–quinolone hybrid that is expected to have activity against MDR *Clostridioides difficile*. The future development of oxazolidinones for human medicine should consider the potential spread of RPP-mediated ARE. The same is true for the development of fusidic acid-based AB that have also gained more attention due to the dramatic increase in FA ARE in recent years, especially among the clinical isolates of *S. aureus* [15]. Current phase 2 clinical trials include sodium fusidate tablets (ARV-1801), granted orphan drug designation by FDA and intended for cystic fibrosis patients with *S. aureus* infections including MRSA. It is not clear if ARV-1801 development accounts for the potential horizontal acquisition of Fus-type RPP genes [36,37,38].

Although macrolides are classified as the highest-priority critically important antimicrobials by the WHO [7] (Table 1), very few viable treatment options are currently available against critical human pathogens due to widespread macrolide (e.g., erythromycin and azithromycin) ARE including the ABC-F RPP-mediated variety. Telithromycin (Ketek) was the first ketolide AB to enter clinical use in 2001 to treat MDR *S. pneumoniae*, among others. However, in 2017, FDA sharply limited its use due to significant safety concerns. Although rare, ketolide (including telithromycin) ARE pathogens have been isolated worldwide having obtained inducible energy-dependent AB efflux and target modification (e.g., *Erm*-mediated rRNA methylation) mechanisms [74]. Resistance to telithromycin in *S. aureus* can be conferred by the ABC-F proteins MsrA, MsrC, and MsrD proteins as well [75]. Although ketolides are a promising class of AB, there are only two candidates currently in clinical trials (Table 3). Solithromycin (Solithera) is in phase 3 and expected to have activity against MDR *Neisseria gonorrhoeae*, and nafithromycin (WCK 4873) is in phase 2. While these new ketolides are not more potent per se than the current ones, they appear not to induce the expression of the corresponding ARE genes to the same degree [23,76,77,78]. However, these new ketolides are less effective when *erm* genes are expressed constitutively [78,79]. On the other hand, solithromycin has been reported to show improved effectiveness against macrolide ARE bacteria (8–16 times more potent than AZM and active against AZM-resistant strains) likely due to its ability to bind to three distinct sites (in contrast to one or two sites in case of the current macrolides) in the bacterial ribosome [80]. Multiple binding sites in the ribosome could render solithromycin less sensitive to RPP (e.g., MsrA, MsrC, and MsrD in *S. aureus*) than telithromycin, but this remains to be determined. Overall, binding to multiple sites in the target is expected to limit ARE development. Furthermore, solithromycin causes less severe side effects, is chemically more stable, and possesses good oral bioavailability compared with earlier ketolides and macrolides. However, to the best of our knowledge, there is no information available about its development for the US market at the moment.

There are currently no new streptogramins, phenicols, or lincosamides in the pipeline. However, it has been reported that the HflXr protein in *Listeria monocytogenes* can mediate ARE to erythromycin as well as to the lincosamide lincomycin [81]. Notably, HflXr rescues translation by splitting and recycling stalled ribosomes in the presence of AB and could therefore be considered a founding member of ribosome protection proteins with novel mechanisms and possible implications for AB design [81].

Taken together, with only one novel translation inhibitor (lefamulin) approved by FDA for clinical use in the last 5 years and none currently in the pharmaceutical pipeline, the treatment options for infections caused by critical MDR pathogens are becoming increasingly dire. While the AB derivatives currently in clinical trials do promise increased efficiency in targeting critical MDR pathogens, the likelihood of approval of a phase 1 candidate has been estimated to be less than 15%. Even when successful, it would take an average of 7 years to reach the market. The FDA and the European Medicines Agency are working on simplification of the approval pathway for AB for selected unmet medical needs. At the same time, some level of cross-resistance and fast adaptation of bacterial populations can be expected, which promises only a short-term solution to MDR. Furthermore, very few oral AB for common diseases associated with high morbidity caused by Gram-negative pathogens are in the pipeline. Therefore, not surprisingly, the WHO has concluded that the current clinical pipeline is insufficient to mitigate the looming ARE threat [59,70]. Clearly, more incentives for investment are needed in the research and clinical development of innovative approaches to overcome ARE in a sustainable manner. Entirely new AB classes, targets, and modes of action are highly desirable to avoid cross- or co-resistance to existing AB classes. According to the WHO’s “Prioritization of pathogens to guide discovery, research and development of new antibiotics for drug-resistant bacterial infections, including tuberculosis,” the situation is especially critical for priority Gram-negative bacteria. These include the carbapenem-resistant pathogens *P. aeruginosa*, *A. baumannii*, and *Enterobacteriaceae* (*Escherichia coli*, *Klebsiella*, fluoroquinolone-resistant *Salmonella*) [59] as strains are emerging worldwide that cannot be treated with any of the AB currently in the market.

### 3.2. Rational Design of Antibiotics

As stressed before, there is an urgent need to address the infections caused by MDR *P. aeruginosa* and other Gram-negative pathogens. ARE in Gram-negative pathogens is mostly exhibited by the carbapenem- and third-generation cephalosporin-resistant phenotype. Notably, the ABC-F RPP proteins (e.g., MsrE) are responsible for macrolide, ketolide, and streptogramin B resistance in clinical *P. aeruginosa* isolates [61] as well as in *A. baumannii*, *A. haemolyticus*, and *K. pneumoniae*, among many other bacteria according to the Comprehensive Antibiotic Resistance Database [29]. High-resolution structures of ARE mechanisms in action can shed light on how existing AB could be improved, or even provide inspiration for new approaches so as to circumvent resistance altogether. Adding novel moieties to existing macrolides (e.g., AZM) or B group streptogramins can improve binding affinity and/or peptidyl transferase inhibition while potentially interfering with the RPP mechanism. AB derivatives with higher affinity for ribosome could compete with ARE ABC-F binding, thereby overcoming resistance. Due to the recent progress in the chemical synthesis of macrocyclic scaffolds, more than 300 structurally diverse macrolide AB candidates were produced, among them drugs that exhibited potent activities against bacterial strains resistant to erythromycin, azithromycin, and other classes of AB [82]. However, while the study included strains with ARE genes such as *ermB* (ribosome methyltransferase) and *mefA* (drug efflux pump), it would be interesting to know how the macrolide candidates fare against pathogens expressing ABC-F proteins. Furthermore, macrolides conjugated with peptides are an option for rational drug design due to variability and the ease of chemical synthesis. Alternatively, ARE ABC-F protein ARD loop mimics are worth consideration for the structure-based drug design of antimicrobial peptides, as they bind in the functionally relevant region of the ribosome and are known to cause conformational changes in PTC key residues [49].

An example of recent structure-guided AB design is the chemically synthesized oxepanoproline scaffold, which, when linked to the aminooctose residue of clindamycin (lincosamide), results in a potent drug (named iboxamycin) with activity against high-priority enterococcal pathogens and a capacity to overcome ARE. Iboxamycin is shown to be effective against pathogens expressing Erm and Cfr methyltransferases and trials in mice are promising [83].

### 3.3. Adjuvants

Antibiotic–adjuvant combination approach is a successful therapeutic strategy and has resulted in several drug entities on the market (e.g., β-lactamase inhibitors that spare β-lactams from hydrolytic destruction) [84,85]. This approach entails the use of bioactive adjuvants that augment the efficacy of AB against ARE pathogens. An adjuvant may be an efflux pump inhibitor, a membrane permeabilizer, or an enzyme inhibitor (e.g., to prevent the degradation of drugs before they reach their targets). ABC-F ATPase inhibitors can serve as potential adjuvants given that ATP hydrolysis is a characteristic requirement for ARE with these RPP. Linezolid and tedizolid are oxazolidinone drugs used to treat infections caused by MDR *Enterococcus* [57]. However, the RPP OptrA has been shown to confer ARE to oxazolidinones and phenicols in *Enterococcus* [53]. Recently, a novel inhibitor of OptrA (CP1) that targets ATPase center was discovered [86]. While this compound forms a weak hydrogen bond with lysine (Lys-271) of OptrA and suppresses its ATPase activity in vitro by only 30%, it highlights the feasibility of designing inhibitors targeting the ATPase center for counteracting RPP-mediated ARE and provide a theoretical basis for further optimization of the candidate structure to obtain inhibitors with higher efficiencies.

### 3.4. Multiple Targets and Combinative Strategies

While over half of the clinically relevant AB target the bacterial ribosome, more attention should be paid to other drug targets as well. In particular, the bacterial cell wall is an excellent target given that it is essential for bacterial survival and growth but is absent from the eukaryotic realm. Furthermore, it is easily accessible to drugs. Indeed, the inhibition of bacterial cell wall synthesis has been exploited for the discovery of highly efficient, broad-spectrum, and safe AB, as exemplified by the success of the β-lactam penicillin [87,88]. The β-lactam ring interacts with the transpeptidase enzyme, disrupting its ability to create peptidoglycan cross links in the bacterial cell wall. However, many bacterial species synthesize β-lactamases that enzymatically cleave the β-lactam ring, rendering penicillins inactive. Methicillin is a modified penicillin derivative that is not a substrate for β-lactamases. Despite this, methicillin-resistant *S. aureus* (MRSA) strains are increasingly common due to the over-prescription of methicillin and related penicillins. *mecA* is responsible for ARE to methicillin and other β-lactam AB. It encodes penicillin-binding protein 2a (PBP2a), which differs from other PBS as its active site does not bind to methicillin or other β-lactams, enabling cell wall synthesis even in the presence of AB. As such, bacterial ARE to β-lactams remains a serious concern to the medical world [87,88]. The bacterial cell wall comprises complex components with immunostimulatory and cytotoxic properties. In addition, it also involves cell-wall-associated adhesion proteins (such as the 1.1 MDa Ebh in *S. aureus* for binding to the extracellular matrix of host cells) [89], and microfibril assembly linked to biofilm formation—all of which could contribute to pathogenesis, infection progression, and ARE. Therefore, investigation of AB action and ARE mechanism involving not only protein synthesis by the ribosome but also the biosynthesis and functioning of the bacterial cell wall, as well as other potential targets, is an interesting field for the development of novel AB and combinative strategies to tackle ARE. Combination therapy could pave the way for an effective solution towards the ARE “crisis.” Hence, structural and functional investigations of the protein synthesis apparatus as well as cell wall biosynthesis and its link to biofilm formation have the potential to address ARE through multiple targets and combinative strategies such as some ongoing projects and recent outcomes in our lab [67,90,91,92,93,94].

## 4. Summary

With the ever-increasing list of multi-drug-resistant pathogens and very few novel antimicrobial agents in the pharmaceutical pipeline, infections that are presently treatable are very likely to become life-threatening once again and affect anyone no matter their age or location. Already, a growing number of infections, such as pneumonia, tuberculosis, blood poisoning, gonorrhea, and foodborne diseases, are becoming more difficult and expensive to treat, as ARE to first-line AB is rising to dangerously high levels in all parts of the world. The CDC estimated that in 2013, more than 2 million people in the US acquired a serious infection from an ARE pathogen and at least 22,000 died as a result. Furthermore, ARE is putting the achievements of modern medicine in peril as organ transplantations, cancer chemotherapy, and even relatively minor surgeries may result in untreatable infections. The WHO names the emergence and spread of ARE among human pathogenic bacteria one of the most complex global health challenges and the biggest impending threat to mankind. The WHO also estimates an additional 1.2 trillion USD of healthcare expenditure per year by 2050 as well as an increase in the economic burden on families and societies. All this underlines the need for the discovery of AB with new scaffolds, uncommon targets, or novel antimicrobial activities. Understanding the ARE mechanisms offers strategic intelligence for the rational improvement of the existing AB classes, as well as helps to inform the development of novel therapeutic approaches to hopefully keep up with the anticipated evolution of ARE. The hot-off-the-press (Crowe-McAuliffe et al., currently preprint in bioRxiv) [95] structural characterization of three (LsaA, Vga_LC_, and VgaL) lincosamide, pleuromutilin, and group A streptogramin resistance ABC-F protein–ribosome complexes isolated by affinity chromatography from clinically relevant pathogens (*Enterococcus faecalis*, *Staphylococcus haemolyticus*, and *Listeria monocytogenes*, respectively) can go a long way in understanding the common yet highly adaptable mechanism shared by ARE ABC-F proteins. ABC-F proteins, which protect the ribosome against a variety of clinically relevant AB in a wide range of human pathogens, are emerging as one of the key players in the global fight against MDR “superbugs”.

## Figures and Tables

**Table 1:** Major classes of protein synthesis inhibitors grouped based on the WHO’s critically important antimicrobials for human medicine as of 2018 [7] with corresponding drug-binding sites, translation inhibition, and antibiotic resistance mechanisms.

Antimicrobial Class	Ribosome Target and Mechanism of Action	Examples of Drugs in Clinical Use	Comments	Resistance Mechanisms
**Highest-Priority Critically Important Antimicrobials**				
**Macrolides and ketolides**	50S NPET-context-dependent modulation of protein synthesis	Azithromycin ^§^	One of few available therapies for serious *Campylobacter* infections and limited theraphy for MDR *Salmonella* and *Shigella* infections. Clarithromycin-resistant Helicobacter pylori causes very common infections in countries of all income levels.	Drug modification/degradation; drug efflux/membrane permeability; target mutation and modification; and **target protection (ABC-F)**
		Clarithromycin ^§^		
		Erythromycin		
		Josamycin		
		Oleandomycin		
		Solithromycin		
		Spiramycin		
		Telithromycin		
		Troleandomycin		
**High-Priority Critically Important Antimicrobials**				
**Aminoglycosides**	30S DC-inhibit translocation and increase error rate	Amikacin *	Sole or limited treatment of MDR tuberculosis and MDR *Enterobacteriacea*	Drug modification/degradation; drug efflux/membrane permeability; target mutation and modification
		Gentamicin *		
		Kanamycin		
		Neomycin		
		Plazomicin ¶		
		Streptomycin		
		Tobramycin		
**Oxazolidinones**	50S PTC (A-site)-context-dependent modulation of protein synthesis (aminoacyl-tRNA binding)	Linezolid ¶	Limited therapy for infections due to MDR *Enterococcus* and MRSA	Drug efflux/membrane permeability; target mutation and modification; and **target protection (ABC-F)**
**Tuberactinomycin**	Subunit interface-inhibit translocation	Capreomycin	Limited theraphy for tuberculosis and other *Mycobacterium* infections	Drug modification/degradation; target mutation and modification
**Highly Important Antimicrobials**				
**Phenicols**	50S PTC (A-site)-context-dependent modulation of protein synthesis (aminoacyl-tRNA binding)	Chloramphenicol *	One of the limited therapies for acute bacterial meningitis, typhoid and non-typhoid fever, and respiratory infections	Drug modification/degradation; drug efflux/membrane permeability; target mutation and modification; and **target protection (ABC-F)**
		Thiamphenicol		
**Lincosamides**	50S PTC (A-site)-inhibit peptide bond formation	Clindamycin *	ARE risk from *Enterococcus* and *Staphylococcus aureus* (including MRSA)	Drug modification/degradation; drug efflux/membrane permeability; target mutation and modification; and **target protection (ABC-F)**
		Lincomycin		
**Steroid antibacterials**	EF-G-inhibit translation elongation and recycling	Fusidic acid	Sole or limited therapy for MRSA infections	Drug efflux/permeability; target mutation; and **target protection (Fus)**
**Streptogramins A (S_A_) and B (S_B_)**	S_A_ 50S PTC (A-and P-sites)-inhibit peptide bond formation; S_B_ 50S NPET-prevent elongation of nascent chain	Dalfopristine (S_A_)	ARE may result from transmission of *Enterococcus* and MRSA from non-human sources	Drug efflux/membrane permeability; target mutation and modification; **target protection (ABC-F)**
		Quinupristine (S_B_)		
**Tetracyclines**	30S DC (A-site)-inhibit delivery of tRNA into A-site	Doxycycline *	Limited therapy for infections due to *Brucella*, *Chlamydia*, and *Rickettsia*	Drug efflux/membrane permeability; drug modification/degradation; target mutation; **target protection (Tet)**
		Tetracycline		
**Important Antimicrobials**				
**Pleuromutilins**	50S PTC (A-and P-site)-inhibit peptide bond formation	Reptamulin	Only used as topical theraphy in humans	Drug efflux/membrane permeability; target mutation and modification; **target protection (ABC-F)**

AB—antibiotic; ABC-F—ATB binding cassette subfamily F proteins; A-site—aminoacyl-tRNA binding site; DC—decoding center; EF-G—elongation factor G; MDR—multi-drug-resistant; MRSA—methicillin-resistant *Staphylococcus aureus*; NPET—nascent peptide exit tunnel; P-site—peptidyl-tRNA binding site; ¶—access group AB [10]; §—watch group AB [10]; *—reserve group AB [10].

**Table 2:** List of ARE ABC-F proteins in pathogens and antibiotic producers with the respective hosts and resistance profiles.

Phylogenetic Lineage	ARE ABC-F in Pathogens and AB Producers	Species	Resistance Phenotype	Drug Binding Site
**ARE 1**	MsrA	*Staphylococcus aureus, Staphylococcus epidermis*	macrolides, ketolides, and group B streptogramins (MKS_B_)	NPET
	MsrC	*Enterococcus faecium*		
	MsrD	*Streptococcus pyogenes, Streptococcus pneumoniae*		
	MsrE	*Pasteurella multocida, Pseudomonas aeruginosa, Escherichia coli*		
	VgaA	*Enterococcus faecalis, Staphylococcus aureus, Staphylococcus haemolyticus*	pleuromutilins, lincosamides, and group A streptogramins (PLS_A_)	PTC A-site overlapping with P-site and NPET
	VgaB	*Staphylococcus aureus*		
	VgaC	*Staphylococcus aureus*		
	VgaD	*Enterococcus faecium*		
	VgaE	*Staphylococcus aureus*		
**ARE 2**	VmlR	*Bacillus subtilis*	pleuromutilins, lincosamides, and group A streptogramins (PLS_A_)	PTC A-site overlapping with P-site and NPET
**ARE 3**	EatA	*Enterococcus faecium*	pleuromutilins, lincosamides, and group A streptogramins (PLS_A_)	PTC A-site overlapping with P-site and NPET
	LsaA	*Enterococcus faecalis*		
	LsaB	*Staphylococcus sciuri*		
	LsaC	*Streptococcus agalactiae*		
	LsaE	*Staphylococcus aureus*		
**ARE 4**	CarA	*Streptomyces termotolerans*	specific to AB produced by each species	PTC A-site overlapping with NPET
	OleB	*Streptomyces antibioticus*		
	SrmB	*Streptomyces ambofaciens*		
	TlrC	*Streptomyces fradiae*		
**ARE 5**	LmrC	*Streptomyces lincolnensis*	specific to AB produced by each species	PTC A-site overlapping with P-site
	VarM	*Streptomyces virginiae*		
**ARE 6**	SalA	*Staphylococcus sciuri*	pleuromutilins, lincosamides, and group A streptogramins (PLS_A_)	PTC A-site overlapping with P-site and NPET
**ARE 7**	OptrA	*Enterococcus faecalis*	oxazolidinones and phenicols (PhO)	PTC (A-site)
**ARE 8**	PoxtA	*Staphylococcus aureus*	oxazolidinones and phenicols (Pho)	PTC (A-site)

A—aminoacyl site; AB—antibiotic; ARE—antibiotic resistance; NPET—nascent peptide exit tunnel; P—peptidyl site; and PTC—peptidyl transferase center. Phylogenetic lineage classification is based on Murina et al. [18,51].

**Table 3:** New bacterial translation inhibitors in clinical use and in the pharmaceutical pipeline.

Name	Class	Developer	Expected Activity Against CDC Urgent or WHO Critical Threat Pathogen	Innovativeness	Comments
**Approved in US since 2017**					
Plazomicin (Zemdri)	Aminoglycoside	Achaogen	CRAB and CRE		WHO’s List of Essential Medicines (see Table 1)
Omadacycline (Nuzyra)	Tetracycline	Paratek	MRSA		
Lefamulin (Xenleta)	Pleuromutilin	Nabriva Therapeutics	MRSA	new chemical class with new mode of action	First pleuromutilin used for systemic treatment of bacterial infections in humans
**Clinical Trial Phase 3**					
Contezolid/contezolid acefosamil	Oxazolidinone	MicuRx Pharmaceuticals Inc.			New drug application submitted (China NMPA)
Solithromycin	Macrolide (ketolide)	Toyama Chemical Co. Ltd.	Drug-resistant *N. gonorrhoeae*		
Eravacycline (Xerava)	Tetracycline	Tetraphase	CRE and MRSA		Granted fast track designation by the FDA
**Clinical Trial Phase 2**					
Nafithromycin	Macrolide (ketolide)	Wockhardt			
ARV-1801 (sodium fusidate)	Fusidic acid	Arrevus Inc.	MRSA		Approved for acute bacterial skin and soft tissue infections in markets outside the US
Delpazolid (LCB01-0371)	Oxazolidinone	LegoChem Biosciences Inc./Nawei Biotechnology			Also in development for tuberculosis treatment
DNV3837/DNV3681	Oxazolidinone-quinolone hybrid	Deinove SA	MDR *Clostridioides difficile*		
**Clinical Trial Phase 1**					
Apramycin (EBL-1003)	Aminoglycoside	Juvabis AG	CRAB and CRE		
TP-271	Tetracycline	La Jolla Pharmaceutical Company	CRAB and MDR *Clostridioides difficile*		No active studies, ongoing out-licensing
TP-6076	Tetracycline	La Jolla Pharmaceutical Company	CRAB and CRE		No active studies, ongoing out-licensing
KBP-7072	Tetracycline	KBP BioSciences Pharmaceutical Technical Co. Ltd.	CRAB		

CDC—Centers for Disease Control and Prevention; CRAB—carbapenem-resistant *A. baumannii*; CRE—carbapenem-resistant *enterococci*; FDA—Food and Drug Administration; MDR—multi-drug-resistant; MRSA—methicillin-resistant *S. aureus*; WHO—World Health Organization.

## Data Availability

Not applicable.

## References

[1] Rodnina M.V., Beringer M., Wintermeyer W. (2006). Mechanism of peptide bond formation on the ribosome. Q. Rev. Biophys..

[2] Arenz S., Wilson D.N. (2016). Bacterial Protein Synthesis as a Target for Antibiotic Inhibition. Cold Spring Harb. Perspect. Med..

[3] Wilson D.N. (2014). Ribosome-targeting antibiotics and mechanisms of bacterial resistance. Nat. Rev. Microbiol..

[4] Poehlsgaard J., Douthwaite S. (2005). The bacterial ribosome as a target for antibiotics. Nat. Rev. Microbiol..

[5] Durand G.A., Raoult D., Dubourg G. (2019). Antibiotic discovery: History, methods and perspectives. Int. J. Antimicrob. Agents.

[6] Forsberg K.J., Reyes A., Wang B., Selleck E.M., Sommer M.O., Dantas G. (2012). The shared antibiotic resistome of soil bacteria and human pathogens. Science.

[7] World Health Organization (2019). Critically Important Antimicrobials for Human Medicine: Ranking of Antimicrobial Agents for Risk Management of Antimicrobial Resistance Due to Non-Human Use. https://www.who.int/foodsafety/publications/antimicrobials-sixth/en/.

[8] Vázquez-Laslop N., Mankin A.S. (2018). How Macrolide Antibiotics Work. Trends Biochem. Sci..

[9] Sothiselvam S., Neuner S., Rigger L., Klepacki D., Micura R., Vázquez-Laslop N., Mankin A.S. (2016). Binding of Macrolide Antibiotics Leads to Ribosomal Selection against Specific Substrates Based on Their Charge and Size. Cell Rep..

[10] World Health Organization (2019). Model List of Essential Medicines. https://www.who.int/groups/expert-committee-on-selection-and-use-of-essential-medicines/essential-medicines-lists.

[11] Jiang M., Karasawa T., Steyger P.S. (2017). Aminoglycoside-Induced Cochleotoxicity: A Review. Front Cell Neurosci..

[12] Choi J., Marks J., Zhang J., Chen D.H., Wang J., Vázquez-Laslop N., Mankin A.S., Puglisi J.D. (2020). Dynamics of the context-specific translation arrest by chloramphenicol and linezolid. Nat Chem. Biol..

[13] Marks J., Kannan K., Roncase E.J., Klepacki D., Kefi A., Orelle C., Vázquez-Laslop N., Mankin A.S. (2016). Context-specific inhibition of translation by ribosomal antibiotics targeting the peptidyl transferase center. Proc. Natl. Acad. Sci. USA.

[14] Tu D., Blaha G., Moore P.B., Steitz T.A. (2005). Structures of MLSBK antibiotics bound to mutated large ribosomal subunits provide a structural explanation for resistance. Cell.

[15] Schlünzen F., Zarivach R., Harms J., Bashan A., Tocilj A., Albrecht R., Yonath A., Franceschi F. (2001). Structural basis for the interaction of antibiotics with the peptidyl transferase centre in eubacteria. Nature.

[16] Gao Y.G., Selmer M., Dunham C.M., Weixlbaumer A., Kelley A.C., Ramakrishnan V. (2009). The structure of the ribosome with elongation factor G trapped in the posttranslocational state. Science.

[17] Seo H.S., Abedin S., Kamp D., Wilson D.N., Nierhaus K.H., Cooperman B.S. (2006). EF-G-dependent GTPase on the ribosome. conformational change and fusidic acid inhibition. Biochemistry.

[18] Wilson D.N., Hauryliuk V., Atkinson G.C., O’Neill A.J. (2020). Target protection as a key antibiotic resistance mechanism. Nat. Rev. Microbiol..

[19] Meydan S., Marks J., Klepacki D., Sharma V., Baranov P.V., Firth A.E., Margus T., Kefi A., Vázquez-Laslop N., Mankin A.S. (2019). Retapamulin-Assisted Ribosome Profiling Reveals the Alternative Bacterial Proteome. Mol. Cell.

[20] Paukner S., Riedl R. (2017). Pleuromutilins: Potent Drugs for Resistant Bugs-Mode of Action and Resistance. Cold Spring Harb. Perspect. Med..

[21] Lin J., Zhou D., Steitz T.A., Polikanov Y.S., Gagnon M.G. (2018). Ribosome-Targeting Antibiotics: Modes of Action, Mechanisms of Resistance, and Implications for Drug Design. Annu. Rev. Biochem..

[22] Peterson E., Kaur P. (2018). Antibiotic Resistance Mechanisms in Bacteria: Relationships Between Resistance Determinants of Antibiotic Producers, Environmental Bacteria, and Clinical Pathogens. Front. Microbiol..

[23] Witzky A., Tollerson R., Ibba M. (2019). Translational control of antibiotic resistance. Open Biol..

[24] Ramu H., Mankin A., Vazquez-Laslop N. (2009). Programmed drug-dependent ribosome stalling. Mol. Microbiol..

[25] Grossman T.H. (2016). Tetracycline Antibiotics and Resistance. Cold Spring Harb. Perspect. Med..

[26] Nguyen F., Starosta A.L., Arenz S., Sohmen D., Dönhöfer A., Wilson D.N. (2014). Tetracycline antibiotics and resistance mechanisms. Biol. Chem..

[27] Jenner L., Starosta A.L., Terry D.S., Mikolajka A., Filonava L., Yusupov M., Blanchard S.C., Wilson D.N., Yusupova G. (2013). Structural basis for potent inhibitory activity of the antibiotic tigecycline during protein synthesis. Proc. Natl. Acad. Sci. USA.

[28] Burdett V. (1991). Purification and characterization of Tet(M), a protein that renders ribosomes resistant to tetracycline. J. Biol. Chem..

[29] CARD 2020 The Comprehensive Antibiotic Resistance Database. https://card.mcmaster.ca/.

[30] Warburton P.J., Amodeo N., Roberts A.P. (2016). Mosaic tetracycline resistance genes encoding ribosomal protection proteins. J. Antimicrob. Chemother..

[31] Connell S.R., Trieber C.A., Dinos G.P., Einfeldt E., Taylor D.E., Nierhaus K.H. (2003). Mechanism of Tet(O)-mediated tetracycline resistance. EMBO J..

[32] Connell S.R., Tracz D.M., Nierhaus K.H., Taylor D.E. (2003). Ribosomal protection proteins and their mechanism of tetracycline resistance. Antimicrob. Agents Chemother..

[33] Dönhöfer A., Franckenberg S., Wickles S., Berninghausen O., Beckmann R., Wilson D.N. (2012). Structural basis for TetM-mediated tetracycline resistance. Proc. Natl. Acad. Sci. USA.

[34] Li W., Atkinson G.C., Thakor N.S., Allas U., Lu C.C., Chan K.Y., Tenson T., Schulten K., Wilson K.S., Hauryliuk V. (2013). Mechanism of tetracycline resistance by ribosomal protection protein Tet(O). Nat. Commun..

[35] Arenz S., Nguyen F., Beckmann R., Wilson D.N. (2015). Cryo-EM structure of the tetracycline resistance protein TetM in complex with a translating ribosome at 3.9-Å resolution. Proc. Natl. Acad. Sci. USA.

[36] Schedlbauer A., Kaminishi T., Ochoa-Lizarralde B., Dhimole N., Zhou S., López-Alonso J.P., Connell S.R., Fucini P. (2015). Structural characterization of an alternative mode of tigecycline binding to the bacterial ribosome. Antimicrob. Agents Chemother..

[37] Olson M.W., Ruzin A., Feyfant E., Rush T.S., O’Connell J., Bradford P.A. (2006). Functional, biophysical, and structural bases for antibacterial activity of tigecycline. Antimicrob. Agents Chemother..

[38] Beabout K., Hammerstrom T.G., Wang T.T., Bhatty M., Christie P.J., Saxer G., Shamoo Y. (2015). Rampant Parasexuality Evolves in a Hospital Pathogen during Antibiotic Selection. Mol. Biol. Evol..

[39] McLaws F.B., Larsen A.R., Skov R.L., Chopra I., O’Neill A.J. (2011). Distribution of fusidic acid resistance determinants in methicillin-resistant Staphylococcus aureus. Antimicrob. Agents Chemother..

[40] Tomlinson J.H., Kalverda A.P., Calabrese A.N. (2020). Fusidic acid resistance through changes in the dynamics of the drug target. Proc. Natl. Acad. Sci. USA.

[41] Tomlinson J.H., Thompson G.S., Kalverda A.P., Zhuravleva A., O’Neill A.J. (2016). A target-protection mechanism of antibiotic resistance at atomic resolution: Insights into FusB-type fusidic acid resistance. Sci. Rep..

[42] O’Neill A.J., Chopra I. (2006). Molecular basis of fusB-mediated resistance to fusidic acid in *Staphylococcus aureus*. Mol. Microbiol..

[43] Borg A., Pavlov M., Ehrenberg M. (2016). Mechanism of fusidic acid inhibition of RRF- and EF-G-dependent splitting of the bacterial post-termination ribosome. Nucleic Acids Res..

[44] Cox G., Thompson G.S., Jenkins H.T., Peske F., Savelsbergh A., Rodnina M.V., Wintermeyer W., Homans S.W., Edwards T.A., O’Neill A.J. (2012). Ribosome clearance by FusB-type proteins mediates resistance to the antibiotic fusidic acid. Proc. Natl. Acad. Sci. USA.

[45] Guo X., Peisker K., Bäckbro K., Chen Y., Koripella R.K., Mandava C.S., Sanyal S., Selmer M. (2012). Structure and function of FusB: An elongation factor G-binding fusidic acid resistance protein active in ribosomal translocation and recycling. Open Biol..

[46] Sharkey L.K., Edwards T.A., O’Neill A.J. (2016). ABC-F Proteins Mediate Antibiotic Resistance through Ribosomal Protection. mBio.

[47] Lenart J., Vimberg V., Vesela L., Janata J., Balikova Novotna G. (2015). Detailed mutational analysis of Vga(A) interdomain linker: Implication for antibiotic resistance specificity and mechanism. Antimicrob. Agents Chemother..

[48] Sharkey L.K.R., O’Neill A.J. (2018). Antibiotic Resistance ABC-F Proteins: Bringing Target Protection into the Limelight. ACS Infect. Dis..

[49] Ero R., Kumar V., Su W., Gao Y.G. (2019). Ribosome protection by ABC-F proteins—Molecular mechanism and potential drug design. Protein Sci..

[50] Fostier C.R., Monlezun L., Ousalem F., Singh S., Hunt J.F., Boël G. (2021). ABC-F translation factors: From antibiotic resistance to immune response. FEBS Lett..

[51] Murina V., Kasari M., Takada H., Hinnu M., Saha C.K., Grimshaw J.W., Seki T., Reith M., Putrinš M., Tenson T. (2019). ABCF ATPases Involved in Protein Synthesis, Ribosome Assembly and Antibiotic Resistance: Structural and Functional Diversification across the Tree of Life. J. Mol. Biol..

[52] Ousalem F., Singh S., Chesneau O., Hunt J.F., Boël G. (2019). ABC-F proteins in mRNA translation and antibiotic resistance. Res. Microbiol..

[53] He T., Shen Y., Schwarz S., Cai J., Lv Y., Li J., Feßler A.T., Zhang R., Wu C., Shen J. (2016). Genetic environment of the transferable oxazolidinone/phenicol resistance gene optrA in *Enterococcus faecalis* isolates of human and animal origin. J. Antimicrob. Chemother..

[54] Hao W., Shan X., Li D., Schwarz S., Zhang S.M., Li X.S., Du X.D. (2019). Analysis of a poxtA- and optrA-co-carrying conjugative multiresistance plasmid from *Enterococcus faecalis*. J. Antimicrob. Chemother..

[55] Sadowy E. (2018). Linezolid resistance genes and genetic elements enhancing their dissemination in enterococci and streptococci. Plasmid.

[56] Antonelli A., D’Andrea M.M., Brenciani A., Galeotti C.L., Morroni G., Pollini S., Varaldo P.E., Rossolini G.M. (2018). Characterization of poxtA, a novel phenicol-oxazolidinone-tetracycline resistance gene from an MRSA of clinical origin. J. Antimicrob. Chemother..

[57] Wang Y., Lv Y., Cai J., Schwarz S., Cui L., Hu Z., Zhang R., Li J., Zhao Q., He T. (2015). A novel gene, optrA, that confers transferable resistance to oxazolidinones and phenicols and its presence in *Enterococcus faecalis* and *Enterococcus faecium* of human and animal origin. J. Antimicrob. Chemother..

[58] Miyoshi-Akiyama T., Tada T., Ohmagari N., Viet Hung N., Tharavichitkul P., Pokhrel B.M., Gniadkowski M., Shimojima M., Kirikae T. (2017). Emergence and Spread of Epidemic Multidrug-Resistant *Pseudomonas aeruginosa*. Genome Biol. Evol..

[59] World Health Organization (2017). Prioritization of Pathogens to Guide Discovery, Research and Development of New Antibiotics for Drug Resistant Bacterial Infections, Including Tuberculosis. https://apps.who.int/iris/handle/10665/311820.

[60] Pereyre S., Goret J., Bébéar C. (2016). *Mycoplasma pneumoniae*: Current Knowledge on Macrolide Resistance and Treatment. Front. Microbiol..

[61] Chen Q., Lu W., Zhou D., Zheng G., Liu H., Qian C., Zhou W., Lu J., Ni L., Bao Q. (2020). Characterization of Two Macrolide Resistance-Related Genes in Multidrug-Resistant *Pseudomonas Aeruginosa* Isolates. Pol. J. Microbiol..

[62] Schroeder M.R., Stephens D.S. (2016). Macrolide Resistance in *Streptococcus pneumoniae*. Front. Cell Infect. Microbiol..

[63] Gentry D.R., McCloskey L., Gwynn M.N., Rittenhouse S.F., Scangarella N., Shawar R., Holmes D.J. (2008). Genetic characterization of Vga ABC proteins conferring reduced susceptibility to pleuromutilins in *Staphylococcus aureus*. Antimicrob. Agents Chemother..

[64] Vimberg V., Cavanagh J.P., Novotna M., Lenart J., Nguyen Thi Ngoc B., Vesela J., Pain M., Koberska M., Balikova Novotna G. (2020). Ribosome-Mediated Attenuation of vga(A) Expression Is Shaped by the Antibiotic Resistance Specificity of Vga(A) Protein Variants. Antimicrob. Agents Chemother..

[65] Lopes E., Conceição T., Poirel L., de Lencastre H., Aires-de-Sousa M. (2019). Epidemiology and antimicrobial resistance of methicillin-resistant *Staphylococcus aureus* isolates colonizing pigs with different exposure to antibiotics. PLoS ONE.

[66] Fan R., Li D., Feßler A.T., Wu C., Schwarz S., Wang Y. (2017). Distribution of optrA and cfr in florfenicol-resistant *Staphylococcus sciuri* of pig origin. Vet. Microbiol..

[67] Su W., Kumar V., Ding Y., Ero R., Serra A., Lee B.S.T., Wong A.S.W., Shi J., Sze S.K., Yang L. (2018). Ribosome protection by antibiotic resistance ATP-binding cassette protein. Proc. Natl. Acad. Sci. USA.

[68] Crowe-McAuliffe C., Graf M., Huter P., Takada H., Abdelshahid M., Nováček J., Murina V., Atkinson G.C., Hauryliuk V., Wilson D.N. (2018). Structural basis for antibiotic resistance mediated by the *Bacillus subtilis* ABCF ATPase VmlR. Proc. Natl. Acad. Sci. USA.

[69] Murina V., Kasari M., Hauryliuk V., Atkinson G.C. (2018). Antibiotic resistance ABCF proteins reset the peptidyl transferase centre of the ribosome to counter translational arrest. Nucleic Acids Res..

[70] World Health Organization (2020). 2020 Antibacterial Agents in Clinical and Preclinical Development. https://www.who.int/publications/i/item/9789240021303.

[71] The Pew Charitable Trusts (2021). List of Antibiotics Currently in Clinical Development. https://www.pewtrusts.org/en/research-and-analysis/data-visualizations/2014/antibiotics-currently-in-clinical-development.

[72] Durães F., Sousa E. (2019). Omadacycline: A Newly Approved Antibacterial from the Class of Tetracyclines. Pharmaceuticals.

[73] Wen Z., Shang Y., Xu G., Pu Z., Lin Z., Bai B., Chen Z., Zheng J., Deng Q., Yu Z. (2020). Mechanism of Eravacycline Resistance in in Clinical *Enterococcus faecalis* Isolates from China. Clinical Front Microbiol..

[74] Georgopapadakou N.H. (2014). The wobbly status of ketolides: Where do we stand?. Expert Opin. Investig. Drugs.

[75] Reynolds E.D., Cove J.H. (2005). Resistance to telithromycin is conferred by msr(A), msrC and msr(D) in *Staphylococcus aureus*. J. Antimicrob. Chemother..

[76] Wang Y., Xiong Y., Wang Z., Zheng J., Xu G., Deng Q., Wen Z., Yu Z. (2021). Comparison of solithromycin with erythromycin in Enterococcus faecalis and Enterococcus faecium from China: Antibacterial activity, clonality, resistance mechanism, and inhibition of biofilm formation. J. Antibiot..

[77] Wen J., Chen F., Zhao M., Wang X. (2019). Solithromycin monotherapy for treatment of community-acquired bacterial pneumonia: A meta-analysis of randomised controlled trials. Int. J. Clin. Pract..

[78] Flamm R.K., Rhomberg P.R., Sader H.S. (2017). Activity of the Novel Lactone Ketolide Nafithromycin (WCK 4873) against Contemporary Clinical Bacteria from a Global Surveillance Program. Antimicrob. Agents Chemother..

[79] Yao W., Xu G., Li D., Bai B., Wang H., Cheng H., Zheng J., Sun X., Lin Z., Deng Q. (2019). Staphylococcus aureus with an erm-mediated constitutive macrolide-lincosamide-streptogramin B resistance phenotype has reduced susceptibility to the new ketolide, solithromycin. BMC Infect. Dis..

[80] Zhanel G.G., Hartel E., Adam H., Zelenitsky S., Zhanel M.A., Golden A., Schweizer F., Gorityala B., Lagacé-Wiens P.R., Walkty A.J. (2016). Solithromycin: A Novel Fluoroketolide for the Treatment of Community-Acquired Bacterial Pneumonia. Drugs.

[81] Duval M., Dar D., Carvalho F., Rocha E.P.C., Sorek R., Cossart P. (2018). HflXr, a homolog of a ribosome-splitting factor, mediates antibiotic resistance. Proc. Natl. Acad. Sci. USA.

[82] Seiple I.B., Zhang Z., Jakubec P., Langlois-Mercier A., Wright P.M., Hog D.T., Yabu K., Allu S.R., Fukuzaki T., Carlsen P.N. (2016). A platform for the discovery of new macrolide antibiotics. Nature.

[83] Mitcheltree M., Pisipati A., Syroegin E.A., Silvestre K.J., Klepacki D., Mason J., Terwilliger D.W., Testolin G., Pote A.R., Wu K.J.Y. (2021). A Synthetic Antibiotic Scaffold Effective Against Multidrug-Resistant Bacterial Pathogens. ChemRxiv.

[84] Douafer H., Andrieu V., Phanstiel O., Brunel J.M. (2019). Antibiotic Adjuvants: Make Antibiotics Great Again!. J. Med. Chem..

[85] Drawz S.M., Bonomo R.A. (2010). Three decades of beta-lactamase inhibitors. Clin. Microbiol. Rev..

[86] Zhong X., Xiang H., Wang T., Zhong L., Ming D., Nie L., Cao F., Li B., Cao J., Mu D. (2018). A novel inhibitor of the new antibiotic resistance protein OptrA. Chem. Biol. Drug Des..

[87] Lobanovska M., Pilla G. (2017). Penicillin’s Discovery and Antibiotic Resistance: Lessons for the Future?. Yale J. Biol. Med..

[88] Sarkar P., Yarlagadda V., Ghosh C., Haldar J. (2017). A review on cell wall synthesis inhibitors with an emphasis on glycopeptide antibiotics. MedChemComm.

[89] Tanaka Y., Sakamoto S., Kuroda M., Goda S., Gao Y.G., Tsumoto K., Hiragi Y., Yao M., Watanabe N., Ohta T. (2008). A helical string of alternately connected three-helix bundles for the cell wall-associated adhesion protein Ebh from *Staphylococcus aureus*. Structure.

[90] Selmer M., Gao Y.G., Weixlbaumer A., Ramakrishnan V. (2012). Ribosome engineering to promote new crystal forms. Acta Crystallogr. D Biol. Crystallogr..

[91] Yu Y., Wu Y., Cao B., Gao Y.-G., Yan X. (2015). Adjustable bidirectional extracellular electron transfer between *Comamonas testosteroni* biofilms and electrode via distinct electron mediators. Electrochem. Commun..

[92] Yang C., Cui C., Ye Q., Kan J., Fu S., Song S., Huang Y., He F., Zhang L.H., Jia Y. (2017). *Burkholderia cenocepacia* integrates cis-2-dodecenoic acid and cyclic dimeric guanosine monophosphate signals to control virulence. Proc. Natl. Acad. Sci. USA.

[93] Ero R., Kumar V., Chen Y., Gao Y.G. (2016). Similarity and diversity of translational GTPase factors EF-G, EF4, and BipA: From structure to function. RNA Biol..

[94] Yan X.F., Xin L., Yen J.T., Zeng Y., Jin S., Cheang Q.W., Fong R.A.C.Y., Chiam K.H., Liang Z.X., Gao Y.G. (2018). Structural analyses unravel the molecular mechanism of cyclic di-GMP regulation of bacterial chemotaxis via a PilZ adaptor protein. J. Biol. Chem..

[95] Crowe-McAuliffe C., Murina V., Turnbull K.J., Kasari M., Mohamad M., Polte C., Takada H., Vaitkevicius K., Johansson J., Ignatova Z. (2020). Structural basis of resistance to lincosamide, streptogramin A, and pleuromutilin antibiotics by ABCF ATPases in Gram-positive pathogens. bioRxiv.

